# Βeta-Cells: Stress, Identity, Failure and Diabetes

**DOI:** 10.3390/cells15050475

**Published:** 2026-03-06

**Authors:** Yousun An, Nicholas Norris, Donglai Li, Jenny E. Gunton

**Affiliations:** 1 Centre for Diabetes, Obesity and Endocrinology, Westmead Institute for Medical Research, University of Sydney, Westmead, NSW 2145, Australia; 2 Faculty of Medicine & Health, University of Sydney, Sydney, NSW 2006, Australia; 3 Department of Diabetes and Endocrinology, Westmead Hospital, Westmead, NSW 2145, Australia

**Keywords:** pancreatic β-cells, type 2 diabetes, obesity, insulin, metabolic stress, ER stress, UPR, dedifferentiation

## Abstract

**Highlights:**

**What are the main findings?**

- Dysfunctional β-cell populations are linked to reduced insulin production and secretion, thereby affecting glucose homeostasis.
- β-cell heterogeneity leads to variable stress-response patterns, thereby influencing the risk of progressive diabetes.

**What are the implications of the main finding?**

- Avoiding glucotoxicity, lipotoxicity and ER stress will aid diabetes prevention and treatment.
- Developing alternative therapies, such as β-cell replacement or anti-diabetic agents that re-active insulin secretion, can open new avenues for diabetes treatment.

**Abstract:**

Type 2 diabetes (T2D) is a pressing global health challenge, primarily driven by modern dietary and lifestyle patterns. Central to T2D progression is the dysfunction of insulin-secreting pancreatic β-cells, which critically disrupts glucose homeostasis. The progression to T2D relies on the β-cells’ inability to compensate for increasing insulin resistance. Initially, β-cells enhance the insulin output, but chronic nutrient overload, ER stress and inflammation ultimately compromise their function and survival. This review examines the molecular and cellular drivers of β-cell failure, focusing on endoplasmic reticulum stress, mitochondrial dysfunction and inflammatory pathways amid chronic metabolic stress. We also explore the loss of β-cell identity and altered interactions within the islet microenvironment. Understanding these mechanisms is essential for developing strategies to prevent β-cell dysfunction and slow T2D progression, ultimately supporting better metabolic health outcomes.

## 1. Introduction

The International Diabetes Federation (IDF) Diabetes Atlas reports that 11.1% of adults, or about 589 million people, have type 2 diabetes (T2D), with 90% of these cases linked to overweight or obesity. Given the rising global prevalence and the substantial costs associated with management and treatment, obesity and T2D together pose significant public health challenges worldwide.

Obesity promotes systemic insulin resistance, consequently increasing the insulin-production burden on pancreatic β-cells [1,2,3]. β-cells compensate for increased insulin requirements by enhancing insulin secretion and expanding the β-cell mass [2,4]. Chronically, metabolic overload and stress can shift compensation toward β-cell dysfunction and failure via multiple mechanisms such as apoptosis and dedifferentiation [5,6,7]. While diabetes is undoubtedly much more common with obesity, a large proportion of people with obesity maintain completely normal glucose tolerance. Therefore, understanding how obesity induces β-cell stress and failure is crucial for clarifying the diabetes pathogenesis and identifying effective therapeutic strategies. This review focuses on mechanisms of β-cell stress, how these mechanisms diminish β-cell function and lead to T2D and highlights emerging molecular targets and treatment approaches to preserve β-cell health.

## 2. β-Cell Stress and Metabolic Overload

The overall β-cell function is driven by both the β-cell mass and the function of β-cells. It is not currently possible to quantify the β-cell mass in a living person. Examining circulating insulin or C-peptide and insulin sensitivity gives an indication of the functional β-cell mass [8]. Individual β-cells contain approximately 20 pg of insulin, stored within roughly 10,000 insulin granules [9,10]. Under physiological conditions, proinsulin is efficiently processed into mature insulin and C-peptide [11]. Nutrient overload can cause metabolic stress in β-cells. The chronic need for hyperinsulinaemia can eventually lead to β-cell exhaustion [12].

**Glucotoxicity, lipotoxicity and gluco-lipotoxicity**

Pancreatic β-cells exposed to hyperglycaemia exhibit an increased insulin release in the short term. If hyperglycaemia persists, there can be compensatory hypertrophy, characterised by an increased β-cell size to meet the insulin demand [4,8]. In rodents, β-cells show a remarkable capacity for adaptive proliferation in response to metabolic stress or pregnancy, which declines in efficiency with age [13,14]. However, human β-cells have a limited proliferative capacity after early childhood [14,15].

If β-cell compensation does not normalise glucose, prolonged exposure to elevated glucose levels can cause toxic effects known as glucotoxicity, further worsening β-cell dysfunction [16,17]. Chronic hyperglycaemia activates multiple stress pathways, including oxidative stress, endoplasmic reticulum (ER) stress [18], increased senescence [19,20] and inflammatory signalling [18]. Excessive mitochondrial reactive oxygen species (ROS) production also increases the oxidation of lipids, proteins and DNA [21]. These mechanisms cause β-cell dysfunction and apoptosis.

Hyperglycaemia stimulates the production of pro-inflammatory cytokines, including interleukin-1β (IL-1β), interferon-γ (IFN-γ) and tumour necrosis factor-α (TNF-α), by various cells, including monocytes/macrophages, T cells and β-cells themselves [22,23,24]. A sustained inflammatory response both contributes to impaired β-cell function, by altering the expression of genes needed for insulin biosynthesis and secretion [25,26,27], and promotes systemic insulin resistance. It also disrupts β-cell function. Recent studies have reported that the deleterious effects of glucotoxicity can spread through exosomes released by islets, transmitting injurious signals to nearby cells [28,29].

Studies identify silent information regulator 1 (SIRT1), a member of the SIRT2 (Sir2)-like family, as a regulator of β-cell function and insulin-responsive tissues [30,31]. SIRT1 activation with resveratrol mitigates the lipid-induced decline in β-cell function in a preclinical study [31]. SIRT1 overexpression in muscle significantly reduces insulin resistance in high-glucose conditions, associated with improvements in the mitochondrial function [30]. Thus, SIRT1 activity is a potential therapeutic target for maintaining glucose homeostasis.

Excess saturated free fatty acids (FFA), such as palmitate, disrupt β-cell homeostasis, in part activating ceramide synthases [32,33]. Accumulated ceramides act as a key mediator of palmitate-induced lipotoxicity in β-cells by increasing ER stress, dysregulated unfolded protein response (UPR) signalling [34], mitochondrial damage and inflammatory pathways [27,35].

ER stress and maladaptive UPR signalling pathways will be discussed, alongside the ER’s function in β-cells, in the following section. Unlike long-term exposure to saturated FFA such as palmitate, monounsaturated fatty acids (MUFA), including oleate, can exhibit protective effects in some β-cell models [36,37]. For example, GPR40, also known as FFA receptor 1 (FFAR1), is a G protein-coupled receptor that modulates insulin secretion in response to fatty acids [38,39] and its activation can yield divergent outcomes. The prolonged activation of GPR40 by MUFA may contribute to protective effects by engaging beneficial signalling pathways that support insulin secretion and promote β-cell survival. Conversely, the chronic and excessive activation of GPR40 is linked to adverse outcomes, impaired β-cell function, hypoinsulinaemia and diabetes [40,41]. The combined excess of both glucose and FFA leads to more severe outcomes, known as glucolipotoxicity [26,32].

Next, we will review the mechanisms underlying stress responses in detail, focusing on their occurrence within organelles and on crosstalk via signalling pathways in the intracellular and extracellular environments in pancreatic islets.

## 3. Mitochondrial Dysfunction in β-Cells

**Figure 1:**
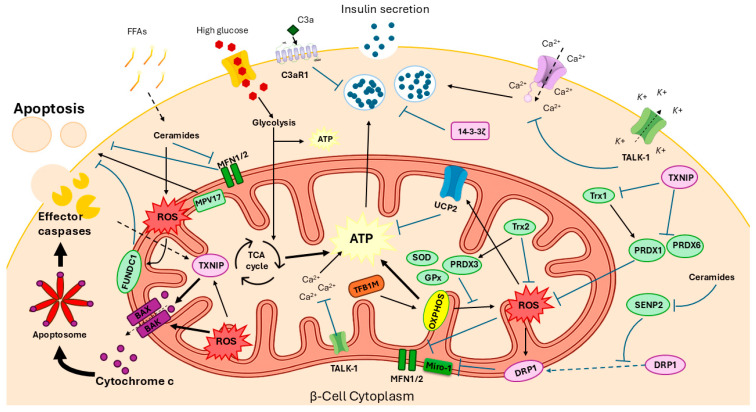
Mechanisms of stress-induced mitochondrial dysfunction and apoptosis in β-cells. Chronic exposure to high glucose and FFAs drives ROS production through excessive oxidative phosphorylation and ceramide accumulation. While the SOD, GPx and PRDX family initially neutralise these reactive species, persistent metabolic stress leads to inactivation of Trx by TXNIP, collapsing the cell’s redox defense. This oxidative stress activates the uncoupling protein UCP2, which alters the proton gradient and reduces ATP production, thereby impairing glucose-stimulated insulin secretion. Ceramides inhibit the mitophagy receptor FUNDC1 and de-SUMOylase SENP2, preventing the clearance of damaged mitochondria and promoting DRP1 recruitment. This disrupts mitochondrial dynamics due to the degradation of MFN1/2 and Miro-1, compounded by TALK-1-mediated alteration of ionic homeostasis (K^+^ and Ca^2+^), which results in a loss of mitochondrial integrity. Collectively, the stress environment triggers BAX/BAK pore formation and cytochrome c release. This initiates the caspase cascade via the apoptosome, ultimately executing β-cell apoptosis.

Here, we review β-cell mitochondrial dysfunction, focusing on key molecular factors (Figure 1). Mitochondria are essential organelles for generating cellular energy via the oxidative phosphorylation of glucose and lipids [42]. Their dysfunction is a pivotal contributor to β-cell impairment and is indicative of β-cell stress response mechanisms and viability [6,7,33]. β-cells have a low antioxidant capacity compared to other cell types [21], which may promote metabolic inflexibility, mitochondrial redox imbalances and ROS accumulation. However, they possess a specialised antioxidant system, involving peroxiredoxin, thioredoxin 1 (TXN1) and thioredoxin reductase (TRXR) to reduce ROS as a defence mechanism [43]. Evidence from in vitro and in vivo studies has identified multiple intracellular factors that are essential for mitochondrial integrity, biogenesis and the insulin secretory capacity in β-cells. Table 1 and Figure 1 present additional information on these factors and their interactions in β-cells.

Mitochondrial calcium (Ca^2+^) homeostasis is critical for β-cell function and disruptions in calcium handling are observed in diabetes. The findings underscore the critical link between the mitochondrial calcium dynamic and β-cell dysfunction in the progression of diabetes. The transcriptome of single islet cells in healthy, pre-diabetic and diabetic mice revealed that Annexin A10 is a candidate pre-diabetic β-cell marker. Under metabolic stress, Annexin 10 is overexpressed, modulating a Ca^2+^ influx, thereby impairing glucose-simulated insulin secretion (GSIS) and leading to β-cell dysfunction [44]. Reduced circulating levels of Reg3g were observed in patients with new-onset diabetes mellitus (NODM) treated with the immunosuppressive medication tacrolimus after heart transplantation. This treatment significantly increases the risk of NODM in these patients. Tacrolimus-induced mitochondrial impairment, including mitochondrial Ca^2+^ uptake, was restored by reintroducing Reg3g [45]. Ca^2+^ channel blockers inhibit short-term insulin release but reduce diabetes risk, mediated in part by their effects on thioredoxin-interacting protein (TXNIP) [46]. Recent research by Graff et al. described a new function of TALK-1, a K^+^-channel which regulates β-cell Ca^2+^ homeostasis. β-cell TALK-1-KO islets exhibited increased mitochondrial calcium and impaired GSIS ex vivo, but mice had improved glucose tolerance both on normal chow and on HFD [47]. This finding suggests TALK-1 is a complex regulator of metabolic remodelling in β-cells and may play a role in the dysregulated glycaemia seen in diabetes.

14-3-3 proteins regulate diverse cellular functions, particularly in metabolism and glucose homeostasis, with significant roles in the β-cell biology [48,49]. Mugabo et al. explored the effects of the 14-3-3 ζ isoform (14-3-3 ζ) in β-cell-specific KO mice and found that the loss of 14-3-3 ζ leads to increased insulin secretion and promotes β-cell proliferation [49].

Several microRNAs, including miR-7, miR-132 and miR-184, play roles in the β-cell physiology by regulating insulin secretion, synthesis and cell survival through controlling genes such as *PDX1* [50,51,52,53]. Using bioinformatic analysis (TargetScan), Cowan et al. explored 16 miRNAs involved in the β-cell function [54]. The study identified miR-29 as a regulator that enhances mitochondrial metabolism and insulin secretion in the rat insulinoma β-cell line, with miR-29 knockdown resulting in defective insulin secretion [54]. Another study examined the effects of miR-146a overexpression in a stable mouse β-cell line and found that overexpression led to mitochondrial dysfunction and enhanced cell death via pro-inflammatory stress responses, whereas its inhibition restored the mitochondrial function and improved insulin secretion [55].

Under metabolic stress, β-cells eliminate damaged mitochondria via mitophagy, an adaptive process that supports β-cell survival [56]. However, prolonged metabolic stress impairs mitophagy, reducing insulin secretion and mitochondrial function. This indicates that the high energy demands of insulin production challenge β-cells, preventing a full recovery of the mitochondrial network [56]. Miro-1 is important for mitochondrial movement and regulates mitophagy by linking mitochondrial dynamics with autophagy. β-cell-specific Miro-1 deletion in mice fed an HFD led to hyperglycaemia and defective mitophagy [57]. FUNDC1 also acts as a mitophagy receptor, aiding the clearance of damaged mitochondria during hypoxia induced by metabolic stress. Specifically, the overexpression of FUNDC1 decreased FA-induced β-cell damage and reduced harmful metabolic markers associated with an HFD in a mouse model [58]. Similarly, NONP1 is an essential mitochondrial matrix protein that modulates mitochondrial proteostasis by degrading misfolded proteins, thereby promoting cell survival under gluco-lipotoxic conditions [59]. Together, these proteins form a regulatory network via regulating mitophagy that β-cells rely on to maintain mitochondrial health, thereby influencing insulin secretion and metabolic resilience.

Given the vital role of the mitochondrial function in β-cell health, recent research has begun exploring innovative ways to restore mitochondrial integrity in β-cells. Zerun et al. evaluated the therapeutic effects of the mitochondrial-targeted nanomedicine Mito-G in an STZ-induced diabetic mouse model [48]. Mito-G accumulates in mitochondria and inhibits Ucp2 activity. In diabetic mice treated with Mito-G every 4 days for 6 weeks while on an HFD, the mitochondrial function is enhanced, mtDNA release is prevented and the STING-mediated autoinflammatory pathway is suppressed, ultimately restoring mitochondrial homeostasis. Together, these results emphasise the need to prevent mitochondrial damage and its associated molecular mechanisms to preserve and enhance β-cell function.

**Table 1:** Effects on mitochondrial function in β-cell lines and animal models.

Molecule; Type	Role in β-Cell Mitochondrial Function	Effects on β-Cells	Model(s)
14-3-3 ζ [49]	14-3-3 inhibition: ↑ OCR, ↑ ATP 14-3-3 ζ KO: ↑ ATP, ↑ mit respiration and OxPhos expression	↑ GSIS, ↑ β-cell proliferation with either inhibition or KO	Mouse islets, β-cells and β-cell KO
C3aR1 GPCR [60]	C3aR1 KO: ↑ stress markers, ↓ MAPK pathway expression and ↓ mit f^n^	↓ GSIS, ↓ β-cell mass, ↑ cell death with lipotoxicity	β-cell-C3aR1 KO mice
Ceramide [35]	↑ Mit membrane permeabilisation and cytochrome C release, ↓ ETC activity	↓ GSIS and ↑ β-cell apoptosis	Rat β-cell lines
CDN1163, SERCA activator [61]	SERCA activation: ↑ mit Ca^2+^ and mit biogenesis. Preserved Ca^2+^ regulation and mit function with FFA	↑ GSIS; improved lipotoxic effect	Mouse β-cell line and islets
Dimt1 [62]	Dimt1 KD: ↓ mit OxPhos expression, ↓ OCR, dissipated mit membrane potential, ↓ ATP	Impaired insulin secretion	Rat, mouse and human β-cell lines
Drp1 Mit fission [63]	Drp1 OE: no changed protein levels of mit respiratory complexes, ↑ fragmented mitochondria	↓ insulin content ↓ GSIS	Mouse β-cell line
Fundc1, Mit membrane [58]	Fundc1 KD: ↓ mit f^n^, ↓ MMP, ↓ ATP, ↑ ROS Fundc1 OE: ↑ mit f^n^, ↑ ATP, ↓ ROS in FA-induced stress	↓ GSIS, ↑ β-cell apoptosis in KD ↑ GSIS, ↓ β-cell apoptosis in OE	Mouse β-cell line
LONP1, Mit protease [59]	Lonp1 KO: ↓ mit f^n^, ↓ mit respiration, ↑ misfolded protein and activation UPR	↓ β-cell mass, ↑ β-cell apoptosis, ↓ GSIS, glucose intolerance	β-cell-KO mice and human islets
Mfn1, Mfn2 Mit fusion [64]	Combined Mfn1/2 KO: ↓ mit respiration, smaller punctate mit structure, ↓ mit DNA content	↓ GSIS ↑ glucose intolerance	β-cell-Mfn1/2 KO mice
Miro-1 Mit GTPase [57]	Miro1 KO: ↓ mitophagy, ↑ damaged mit, ↑ ROS and inflammatory responses under HFD stress	↓ insulin signalling via IRS-Akt-FoxO1, ↓ insulin secretion, ↑ glucose intolerance with HFD	β-cell-Miro1 KO mice
miR-146a-5p [55]	OE: ↑ mit depolarisation, ↓ mit function markers KD: ↑ mit f^n^, DNA copy number, respiration, ATP	OE: ↑ β-cell death KD: ↑ insulin secretion	Mouse β-cell line
Mpv17, Mit membrane [65]	No affected ROS and mit DNA levels, ↓ caspase-3 activation in response to STZ or FA	↓ β-cell apoptosis in response to STZ or FA	Mpv17 KO mice and β-cell line
Pgc-1a [66]	Pgc-1a KO: small effects on mit gene expression and β-cell mass, function	↓ GSIS ↑ lipid in KO islets	β-cell-Pgc-1a KO mice
Prdx6, Antioxidant enzyme [67]	Prdx6 KD: ↓ ATP, ↓ intracellular Ca^2+^, ↓ mit volume and mass, ↓ mit potential and OCR	↓ insulin secretion ↑ β-cell apoptosis	Prdx6 KD mouse β-cell line
Reg3g [45]	Reg3g stimulation: restored mit function via ↑ MMP, mit Ca^2+^ uptake, ATP production and OCR	↑ GSIS and β-cell regeneration	Mouse islets, mouse and human cell lines
Senp2 Protease [68]	Senp2 KO: ↑ mit dysfunction via reduced active form of Drp1, enlarged mit and lower OCR	↓ GSIS and impaired GTT	β-cell-KO mice and β cell line
Talk1, ATP-K^+^ channel [47]	Talk1 KO: ↑ mit Ca^2+^, ↑ mit ATP production ↓ ATP-linked respiration, ↓ OCR with HFD	Improved GTT, no change in effect on GSIS under HFD stress	β-cell–Talk-1 KO mice
Tfb1m [69]	Tfb1m KO: ↓ mit ATP production via perturbed OxPhos. Disrupted mit ultrastructure	↑ β-cell death via β-cell apoptosis and necrosis, ↑ inflammation	β-cell-Tfb1m KO mice
Txnip, Redox regulator [70]	Inhibition with SRI-37330 restored redox balance and ↓ ROS in response to FA	↑ β-cell f^n^, ↑ GSIS, ↑ ER redox state and proinsulin trafficking	FA-cultured rat β-cell line
Ucp2 [71]	Inhibition: ↓ IL-1β activity, ↓ mit DNA and leaking in Ucp2 inhibitor, Mito-G	↑ insulin secretion, ↑ β-cell mass, ↓ β-cell death	Mice, STZ, db/db β-cell lines
Zfp148 [72]	Zfp148 KO: ↑ mit PEP-dependent Ca^2+^ influx	↑ mit PEP-dependent insulin secretion	β-cell–Zfp148 KO mice

Abbreviations: ↑, increase; ↓, decrease; ATP, adenosine triphosphate; C3aR1, Complement C3a Receptor 1; CDN1163, allosteric sarco/endoplasmic reticulum Ca^2+^-ATPase (SERCA) activator; db/db, genetically diabetic-obese rodents; Dimt1, Dimethyl-adenosine transferase 1; Drp1, Dynamin-Related Protein 1; ER, Endoplasmic Reticulum; ETC, mitochondrial electron transport chain; FA, fatty acids; F^n^, function; Fundc1, FUN14 domain-containing protein 1; GTT, glucose-tolerance test; GSIS, glucose-stimulated insulin secretion; HFD, high-fat diet; KD, knockdown; KO, knockout; Lonp1, Lon Peptidase 1; Mfn1, Mitofusin-1; Mfn2, Mitofusin-2; Mit, mitochondrial; Mpv17, Mitochondrial inner membrane protein Mpv17; Miro1, Mitochondrial Rho GTPase 1; MMP, mitochondrial membrane potential; OCR, oxygen consumption rates; OE, overexpression; OxPhos, mitochondrial oxidative phosphorylation; PEP, phosphoenopyruvate; Prdx6, peroxiredoxin 6; Pgc-1a, Peroxisome proliferator-activated receptor-γ coactivator 1-α; Reg3g, Regenerating Islet-derived protein 3 γ; ROS, reactive oxygen species; Senp2, Sentrin-specific protease 2; STZ, streptozotocin; TALK-1, TWIK-related alkaline pH-activated K^+^ channel 1; Tfb1m, Transcription Factor B1; Txnip, thioredoxin-interacting protein; Ucp2, uncoupling protein 2; Zfp148, zinc finger protein 148.

## 4. Maladaptive UPR During Chronic ER Stress in Pancreatic β-Cells

**Figure 2:**
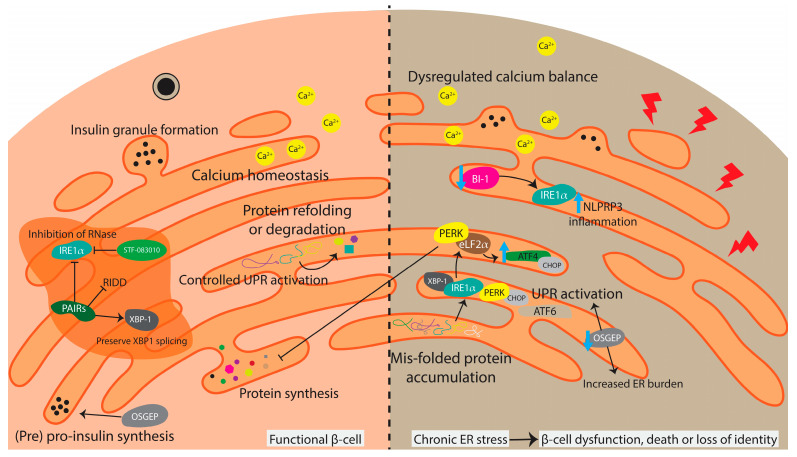
ER quality control and UPR signalling determine β-cell fate under metabolic stress. Schematic illustrating the balance between adaptive (**left**) and maladaptive ER responses (**right**) in β-cells [75]. Under physiological conditions (**left**), efficient ER quality control, including OSGEP-dependent proinsulin folding, balanced Ca^2+^ homeostasis and controlled activation of UPR sensors, supports proper insulin synthesis, granule formation and glucose homeostasis. Under ER stress (**right**), misfolded protein accumulation, Ca^2+^ dysregulation and loss of ER quality control lead to hyperactivation of UPR.

This section aims to describe the mechanisms of the UPR, emphasising recent findings and exploring potential therapeutic strategies derived from these mechanisms, particularly regarding stress responses in the ER within β-cells (Figure 2). In pancreatic β-cells, the requirement for substantial insulin synthesis in the ER places a high demand on the ER function [10]. Proinsulin is translated in the ER, where it undergoes folding, glycosylation and the cleavage of C-peptide to ensure proper insulin protein conformation [73,74]. The ER features a quality control system to identify misfolded proteins for refolding or degradation, vital for cellular health [73,74]. When misfolded or unfolded proteins accumulate in the ER, the UPR is activated.

Stressors such as imbalanced nutrient levels or altered oxygen tension increase misfolded protein accumulation in the ER, triggering the UPR through three signalling pathways mediated by Inositol-Required Enzyme 1 α (IRE1α), PKR-like ER kinase (PERK) and Activating Transcription Factor 6 (ATF6) [76,77]. In β-cells, a high insulin demand can stress-activate the UPR. A ‘successful’ UPR slows protein synthesis to address the build-up of misfolded proteins. Under prolonged ER stress, the UPR shifts from a protective to a maladaptive state, primarily through sustained IRE1α–XBP1 and PERK–CHOP signalling [78,79]. Continued IRE1α activity leads to excessive mRNA degradation, further promoting stress signalling driven by JNK and NF-κB, which mediates inflammatory gene expression [80,81]. Meanwhile, persistent PERK–eIF2α signalling reduces the overall protein translation while increasing ATF4-driven CHOP expression, causing oxidative stress and the dysregulation of the ER–mitochondrial calcium balance, ultimately leading to β-cell death [82,83]. Overwhelming the UPR’s compensatory capacity due to the duration and/or intensity of the stimulus causes β-cell failure and death [84].

A recent study by Yujie et al. explored O-sialoglycoprotein endopeptidase (OSGEP) as an important factor in ER quality control, including proinsulin folding and UPR activation [85]. β-cell-specific OSGEP deletion resulted in abnormal glucose homeostasis and hypoinsulinaemia, particularly under HFD conditions. An OSGEP deficiency impaired proinsulin folding, reducing the insulin translational efficiency. Gene profiling found changes in UPR and apoptosis-related gene expression, indicative of increased ER stress [85]. Exposure to metabolic stressors, such as an HFD or ER stress inducers, further increased UPR signalling molecules, including IRE1α, ATF6 and CHOP (Figure 2). These findings suggest that OSGEP stimulation has potential as a therapeutic target to preserve the β-cell function.

Feldman et al. reported that modulating IRE1α activity using their Partial Antagonists of IRE1α RNase (PAIRs compound) preserved XBP1 splicing while suppressing potentially deleterious mRNA products via Regulated IRE1-Dependent Decay (RIDD) [86]. PAIRs treatment was associated with increased insulin mRNA levels and elevated proinsulin proteins [86]. Managing higher proinsulin production demands significant cellular resources, which may lead to β-cell fatigue and damage. However, despite the higher level of proinsulin, PAIRs treatment reduced apoptosis in β-cells and RNA-Seq profiling revealed the attenuation of pro-apoptotic gene expression with IRE1α overexpression [86].

Bax Inhibitor-1 (BI-1) is also a negative regulator of IRE1α activity, showing positive effects on insulin-producing β-cells and glucose metabolism [87]. Mice lacking BI-1 showed early signs of diabetes with excessive IRE1α RNase activity and ER stress, ultimately leading to β-cell failure on an HFD through the activation of β-cell death signalling and NLRP3-mediated inflammatory responses [87]. After the induction of β-cell dysfunction with an HFD, treatment with STF-083010, an inhibitor of IRE1α RNase activity, reversed the metabolic phenotype, reducing ER stress and restoring the insulin granule content [87].

In compensatory ER stress responses, IRE1α-mediated pathways enhance the function of β-cells [88]. Exogenous ER-degradation-enhancing α-mannosidase-like protein 1 (EDEM1) helps to mitigate excess IRE1α cascades, increasing the insulin levels in diabetic rats and insulinoma β-cells [88]. Given these findings, IRE1α-mediated UPR signalling pathways may present a promising strategy for diabetes treatment.

The analysis of pancreatic tissue from living donors, including normal glucose-tolerant individuals, glucose-intolerant individuals and T2D patients, revealed alterations in the islet gene expression profiles [89]. With an increasing glucose intolerance, there was a progressive increase in the expression of ER stress-related genes, such as *PDIA1*, *XBP1* and *GRP78*, and a corresponding decrease in functional islet phenotype-related genes, such as *MAFA*, *NKX6.1* and *CCT4*, in glucose-intolerant and T2D groups, indicating accelerated ER stress, defective insulin synthesis and a loss of β-cell identity in living individuals [89], consistent with preclinical and experimental cellular studies [90]. However, reduced levels of active XBP1 protein (spliced XPB1) were observed in both animal models and T2D patients [91]. These conflicting characteristics in XBP1 mRNA and protein levels may result from an insufficient adaptive response to ER stress during disrupted glucose homeostasis in T2D patients, or from decreased XBP1 protein synthesis and stability, leading to lower active XBP1 protein levels. Several biotechnology tools are developing UPR modulators and have filed patents, but clinical trials assessing their safety and efficacy in diabetic patients are still limited or absent. This gap highlights both the translational potential and the need for further research on UPR-targeted therapies for diabetes.

The ER also synthesises lipids essential for cellular membranes and insulin granules and serves as a crucial calcium reservoir, noting that calcium fluctuations play a key role in insulin exocytosis [83,92]. Calcium re-uptake in the ER is mediated via the SERCA proteins. Given the central role of the ER in calcium homeostasis, protein folding and granule biogenesis, perturbations in ER calcium handling can rapidly induce ER stress and activate the UPR. Importantly, ER stress does not occur in isolation, but is tightly integrated with mitochondrial function through dynamic ER-mitochondrial crosstalk.

UPR/ER/mitochondrial crosstalk

Crosstalk between UPR signalling pathways in the ER and mitochondrial response mechanisms upon stress warrants further investigation. The interaction between the ER and mitochondria regulates the cellular redox balance and apoptotic signalling, but under conditions of stress can amplify oxidative stress and cell death pathways. Mitochondrial dysfunction disrupts the redox cycles of the ER, leading to increased hyper-oxidation within the ER and delays in proinsulin export [70]. This restricts insulin granule formation and negatively affects glucose metabolism. TXNIP suppression via a pharmacological inhibitor, SRI-37330, led to restored ER redox homeostasis and improved insulin secretion in cultured β-cells [70]. These findings offer important insights into how the dysfunction and regulation of both mitochondria and the ER jointly contribute to disturbances in glucose metabolism.

Chronic stress may ultimately culminate in β-cell senescence. Importantly, chronic stress-driven mitochondrial–ER crosstalk induces β-cell senescence, a process well known to involve dysregulated cell-cycle control via p16 and p21 [93]. This, in turn, leads to a senescence-associated secretory phenotype (SASP), which enables the production of pro-inflammatory cytokines and chemokines, causing paracrine inflammation and impairing neighbouring β-cell function [94]. A single-cell transcriptomic study of human islets has also identified senescence-associated gene expression signatures in individuals aged between 4 months and 75 years [95]. Although this study was conducted in non-diabetic humans, these findings emphasise the translational relevance of targeting senescence pathways to preserve β-cell function and suggest therapeutic approaches for diabetes prevention. In this context, senolytics, which target senescent cells and induce apoptosis via SASP-mediated signalling, have been evaluated as therapeutic candidates to remove senescent β-cells and have been examined in preclinical models and early-phase human clinical trials [96]. Senolytic-treated clinical pilot studies in diabetes and other diseases, especially age-related disorders, have indeed revealed the enhanced apoptosis of senescent cells, along with SASP-mediated signalling, including elevated levels of senescence-associated secreted proteins and cytokine–cytokine receptor interactions [97]. This has not been tested in human diabetic islets. Consequently, this can improve the neighbouring β-cell function and overall islet integrity. However, large-scale studies and long-term assessments are required to obtain approval for human clinical trials of the medication, given potential adverse effects such as gastrointestinal discomfort, weakness and impaired wound healing.

## 5. β-Cell Identity and Dedifferentiation

**Figure 3:**
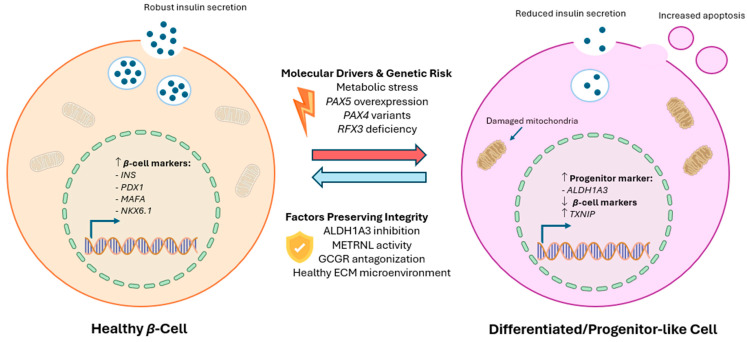
Mechanisms of β-Cell plasticity, dedifferentiation and potential therapeutic reprogramming. Under chronic metabolic stress, mature β-cells may undergo dedifferentiation, losing their identity and reverting to a progenitor-like or immature state. This process is non-linear and can be reversible. Key genetic factors influence this trajectory, including *PAX5* overexpression, *RFX3* deficiency drives dysfunction and apoptosis and *PAX4* variants (e.g., Arg192His) increase T2D susceptibility by reducing insulin content. Conversely, METRNL maintains β-cell integrity by preventing transdifferentiation. Antagonism of the glucagon receptor and inhibition of *ALDH1A3* promote redifferentiation. Additionally, iPSC-derived islet organoids, supported by specialised ECM hydrogels, could restore glucose homeostasis.

Pancreatic β-cells are dynamic, exhibiting plasticity, functional adaptation and cellular reprogramming in response to metabolic demands. However, chronic metabolic stress can drive β-cells toward functional dedifferentiation, contributing to β-cell failure. This is characterised by reprogramming, with the reduced expression of the key β-cell identity and maturity markers, such as *INS*, *PDX1*, *MAFA* and *NKX6.1*, alongside the reactivation of progenitor-like or immature transcriptional programs [98,99,100,101,102,103,104,105]. Interestingly, repeated or episodic stress can similarly overwhelm the adaptive capacity and lead to the loss of the β-cell identity [84]. In addition to β-cell identity genes, XBP1 also contributes to the identity of functional β-cells and prevents transdifferentiation into glucagon-expressing α-cells under metabolic stress conditions [90]. Figure 3 summarises β-cell plasticity, dedifferentiation and therapeutic reprograming.

T2D is a polygenic disease influenced by the interplay among multiple genetic variants and environmental factors [106,107,108,109]. Thanks to advanced genetic tools and large-scale cohort studies, including genome-wide association studies (GWAS) [110,111], meta-analyses [112,113] and next-generation sequencing (NGS), researchers have identified genetic risk factors associated with T2D, such as TCF7L2 [114,115], SLC30A8 [116,117] and KCNJ11 [118,119]. These are major risk factors that contribute to dedifferentiation by disrupting the transcriptional system, insulin granule function and GSIS, compromising the β-cell identity and function. Both common and rare variants have been integrated into gene regulatory networks across large populations. Building on this knowledge, this section reviews studies from the past five years focusing on genes involved in β-cell dedifferentiation. We will also discuss emerging strategies to promote β-cell redifferentiation toward an insulin-producing phenotype.

A study by Zhou et al. found that the meteorin-like (METRNL) protein is a regulator of β-cell integrity under metabolic stress, preventing β- to α-cell transdifferentiation [120]. Specifically, βKO-METRNL mice exhibited impaired GSIS, the activation of the C-Jun-mediated stress program and suppression of genes linked to the β-cell identity [120]. Single-cell RNA sequencing analysis revealed that these mice developed non-β-cell transcriptional signatures such as the upregulation of α-cell genes (*Gcg*, *Irx2* and *Arx*) but downregulation of β-cell genes (*Ins1*, *Ins2 MafA* and *Pdx1*). Another study by Cui et al. in diabetic mouse models showed that antagonising the glucagon receptor (GCGR) with a monoclonal antibody (mAb) promotes the transdifferentiation of pancreatic α-cells into β-cells [121]. Within the islet cells of the pancreas, the GCGR is predominantly expressed in β-cells, while its expression is low in α-cells and negligible in δ-cells [122,123]. Experiments with isolated mouse islets revealed significantly enhanced insulin secretion under GCGR mAb culture conditions, with the upregulation of genes associated with β-cell integrity and regeneration, including *Gcg*, *Pcsk1*, *Ins1*, *Pdx1* and *Ngn3* [121]. Together, these findings suggest that GCGR inhibition may counteract β-cell dedifferentiation by reinforcing β-cell fate commitment.

RFX3, a member of the regulatory factor X family of transcription factors, serves as a central regulator of developmental gene expression, particularly during primary ciliogenesis and neuroendocrine lineage specification [106,107]. These functions were also observed in a human induced pluripotent stem cell (hiPSC)-based study, which demonstrated that *RFX3* is essential for stabilising the islet endocrine cell identity and promoting β-cell differentiation and function [107]. Notably, an *RFX3* deficiency led to the upregulation of TXNIP and β-cell failure via apoptosis under stress conditions [107]. Collectively, these findings suggest that RFX3 functions as a transcriptional integrator associated with islet developmental signalling, stress adaptation and β-cell vulnerability.

Using approximately 300 human pancreatic islet samples, gene expression analysis identified candidate genes associated with T2D risk, including *PAX5* [108]. The overexpression of PAX5 in β-cells caused severe mitochondrial dysfunction, impacting β-cell function, and regulated T2D-associated genes [108]. Recent research using human β-cells from East Asian populations showed that the Arg192His coding variant of *PAX4* is associated with T2D susceptibility [109,110]. The hiPSC-derived β-like cells carrying the Arg192His or Tyr186X variants expressed lower *PAX4* along with a reduced insulin content [110]. The CRISPR-based gene correction of the Arg192His or Tyr186X alleles restored the β-cell identity and total insulin content in hiPSC-derived β-like cells [110]. Collectively, these findings highlight *PAX4* and *PAX5* as important regulators of β-cell identity.

Most research relies on preclinical studies using β-cell-specific gene-edited rodents or in vitro cellular experiments to identify genes linked to β-cell integrity in islets and T2D-associated genetic variants. Human studies usually utilise post-mortem pancreatic islets from individuals with advanced T2D. A recent study applied a multi-omics analysis of pancreatic islets from living metabolically characterised individuals with diabetes [111]. People in the study had a planned pancreatectomy due to chronic pancreatitis (~25%, type 3C diabetes) or pancreatic tumours (type 2 diabetes). It demonstrated that β-cell heterogeneity increases from the prediabetic stage and that β-cell remodelling is non-linear [111]. These results suggest that β-cells do not decline uniformly, but may exist in dynamic states that can be remodelled in response to metabolic stress.

Indeed, human single-cell multi-omics studies have emphasised β-cell heterogeneity and stress dynamics in response to the surrounding environment, revealing reversible state transitions rather than static states. Integrated single-cell RNA-seq, single-nuclei ATAC-seq and 3D genome-wide profiling identify β-cell subpopulations with distinct stress resilience. These findings indicate that β-cell populations are heterogeneous, comprising both immature and mature phenotypes rather than being uniformly damaged. Thus, β-cells show high insulin expression, consistent with a compensatory response, and low insulin expression, which may reflect functional impairment or dedifferentiation. Moreover, a distinct subcluster within β-cell populations shows the activation of inflammatory pathways and ER stress/UPR, along with linked genes, suggesting subtypes that may be particularly vulnerable under chronic metabolic stress—the transcription factors, such as HNF1A, are key drivers of heterogeneity and functional differences [112].

A recent single-cell multi-omics study found that β-cells in T2D show gender-specific transcriptional and epigenomic alterations, with mitochondrial respiration suppression enriched in females and insulin-secretion signatures more prevalent in males [113]. Moreover, T2D-associated genetic variants preferentially localise to sex- and cell-type-specific regulatory chromatin regions, suggesting that β-cell stress vulnerability is differentially modulated by sex [113]. Beyond β-cells, another single-cell multi-omics study utilising imaging mass cytometry (IMC) revealed significant alterations in the pancreatic islet architecture, islet cell subtypes (including α- and β-cell composition) and immune cells (including CD8 T cells and macrophages) compared with healthy individuals and those with T2D [124]. Furthermore, IMC enhances the visualisation of islet heterogeneity, subtype ratios and cell–cell interactions in pancreatic tissue [124]. This approach can help reveal the β-cell stress response and can be utilised as a potential diagnostic indicator of prediabetes and diabetes through integrated single-cell multi-omics analysis.

A recent study identified *ALDH1A3* as a factor in both β-cell dysfunction in T2D and β-cell dedifferentiation [125]. Lineage-tracing experiments showed that *ALDH1A3*-dedifferentiated β-cells can be reconverted into functional, mature β-cells, illustrating that β-cell dedifferentiation can be reversible [125]. The inhibition of *ALDH1A3* via genetic or pharmacological tools restored insulin secretion and promoted differentiation and regeneration [125], underscoring the therapeutic potential of targeting β-cell plasticity. Recent studies highlight the potential roles of stem-cell-derived β-cell differentiation and islet organoids containing insulin-producing β-like cells as a novel therapeutic vision for T2D [114]. The microenvironment is important for function in iPSC-derived islet organoids [115]. Using a decellularised amniotic membrane ECM hydrogel with collagen VI, laminin and fibronectin, Zhu et al. created islet organoids and then transplanted them into diabetic mice, resulting in improved glucose tolerance [115]. The results indicate that while encouraging β-cell differentiation is key, supporting the interactions between islets and their environment is also crucial for in vivo glucose control.

## 6. Inflammation and Stress Responses

Insulin-producing pancreatic β-cells are both initiators and victims of inflammatory responses under stress conditions [116]. In addition to responding to immune cell signals, β-cells shape the inflammatory milieu of the islet microenvironment [117,118]. β-cell inflammatory signalling can dynamically shift from an adaptive, protective response to pathological, self-perpetuating inflammation, leading to the progression of β-cell failure [117,119]. As described above, prolonged stress impairs the β-cell mitochondrial function and increases ROS and mitochondrial DNA damage, which activates inflammatory pathways. This activation can occur independently of immune cell-derived cytokines [126,127].

The activation of the NLRP3 inflammasome is also responsible for the pro-inflammatory responses, leading to the cell death observed in diabetes [128]. In pancreatic β-cells, chronic hyperglycaemia enhances the expression of TXNIP, promoting NLRP3 inflammasome activation. The TXNIP/NLRP3 inflammasome axis leads to caspase-1 activation. Activated caspase-1 cleaves gasdermin D (GSDMD) at its N-terminus. The N-terminal fragment binds to the plasma membrane and builds pores. Through these pores, pro-inflammatory cytokines such as IL-1β and IL18 are rapidly released, resulting in pro-inflammatory-formed cell death (pyroptosis) [128,129]. Modulating inflammatory responses within the islet microenvironment could be beneficial for β-cell integrity. The TXNIP-NLRP3-caspase-1/GSDMD axis is thus a therapeutic target for blocking inflammasome-induced β-cell loss and may slow the progression of diabetes. Improved outcomes with the inhibitor MCC950, targeting the TXNIP/NLRP3 inflammasome axis to treat inflammatory and metabolic disease, were observed in preclinical studies. Notably, the administration of MCC950 improved the glycaemic control in islet-transplanted mice, a finding strongly linked to reduced islet death via IL-1β suppression, especially supporting long-term graft islet survival [130]. Future research is also required to determine whether MCC950 can produce synergistic effects when combined with existing anti-diabetic treatments and whether it can also have positive effects on related complications beyond diabetes.

A study by Grieco et al. highlighted a critical role for miR-184-3p in regulating CREB-regulated transcription coactivator 1 (CRTC1) within β-cell-intrinsic inflammatory signalling [131]. In islets from patients with T2D, miR-184-3p was reduced, resulting in the upregulation of its target *CRTC1* [131]. Increased CRTC1 protects β-cells from palmitate- and cytokine-induced apoptosis, indicating an adaptive survival response under stress. Notably, reduced miR-184-3p levels were associated with reductions or the cytoplasmic translocation of the β-cell identity transcription factor NKX6.1. NKX6.1 positively regulates miR-184-3p expression and is linked to the β-cell identity and functional capacity. This study suggests the reduction in miR-184-3p and resulting upregulation of CRTC1 in T2D is followed by the reduction or translocation of transcription factor NKX6.1 seen within dysfunctional or dedifferentiated β-cells in T2D. NKX6.1 is closely linked to β-cell identity loss, functional decline and dedifferentiation. These findings suggest that the miR-184-3p-CRTC1 axis serves a compensatory role, allowing β-cells to evade apoptosis at the cost of β-cell identity and function.

In contrast, the accumulation of human islet amyloid polypeptide (hIAPP) in T2D is closely linked to amyloid deposition, inflammation and a loss of β-cells [132]. A study identified Receptor-Interacting Protein Kinase 3 (RIPK3) as a driver of amyloid production and potential β-cell damage [132]. This study explored RIPK3’s role in amyloid-driven β-cell loss and inflammation in mice transgenic for hIAPP [132]. Macrophages were the main source of RIPK3 in islets; depleting RIPK3 in bone-marrow-derived macrophages (BMDMs) resulted in lower inflammatory gene expression, including *Tnfa*, *Il1b* and *iNos* (inducible nitric oxide synthase) [132]. hIAPP mice fed an HFD had glucose intolerance and a loss of β-cell mass which was ameliorated with RIPK3 knockout [132]. In this context, a study by Chen et al. provided insights into how interactions with microenvironmental components, such as macrophages within the islet, affect insulin secretion in obese diabetic animals [119]. They demonstrated that macrophage-mediated IL-6 signalling is important for GSIS in lean mice, but this pathway is impaired in T2D patients and obese diabetic mice [119]. An impaired FFA receptor 4 (FFAR4)-mediated IL-6 release and reduced downstream signalling were observed in these animals [119]. Exogenous IL-6 treatment restored GSIS [119].

Recent studies have proposed a beneficial anti-inflammatory role for butyrate, which protects β-cells from cytokine-induced stress and functional impairment through IL-1β-signalling pathways. The treatment of mouse islets and a rat insulinoma β-cell line with butyrate significantly reduced the IL-1β-induced expression of *Nos2*, *Cxcl1* and *Ptgs2*, as well as iNOS production [133]. Butyrate did not affect the nuclear translocation of NF-κB p65, but did promote the acetylation of NF-κB p65 and histone H4, suggesting that the suppression of inflammatory gene transcription may be mediated by histone deacetylase (HDAC) inhibition [133]. A study from the same group used a genome-wide functional analysis of H3K27 acetylation (H3K27ac) to identify an epigenetic mechanism by which butyrate restores IL-1β-reduced H3K27ac levels at loci associated with hormone secretion [134]. This redistribution of H3K27ac allows a transcriptional reprogramming process through which butyrate modifies the β-cell epigenetic landscape [134]. Overall, modulating inflammatory responses in β-cells are likely to maintain β-cell health.

## 7. Summary and Future Directions

**Figure 4:**
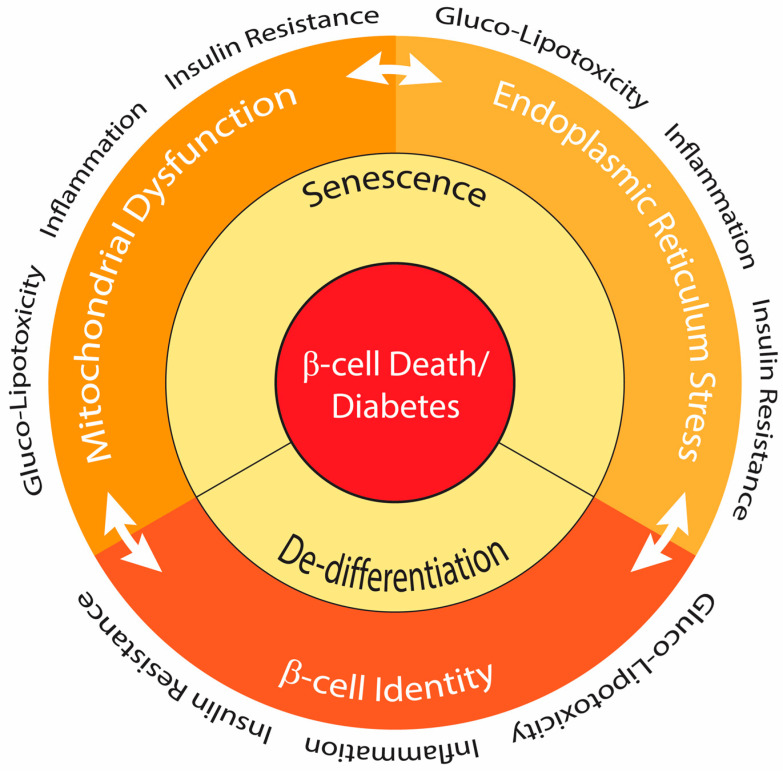
Integrated stress networks driving mitochondrial dysfunction, ER stress, β-cell senescence and identity loss in response to metabolic stress associated with diabetes. Metabolic stressors linked to diabetes such as glugo-lipotoxicity, inflammation and insulin resistance interact closely to induced ER stress, mitochondrial dysfunction and β-cell dedifferentiation. These processes reinforce each other, leading to cell senescence and loss of cell identity, ultimately resulting in progressive β-cell failure and diabetes.

In conclusion, the complex interplay of multifactorial stress pathways affecting insulin-producing β-cells is central to the development of β-cell failure in obesity and T2D. This failure occurs when cumulative stress networks exceed the capacity of adaptive mechanisms (Figure 4).

Preclinical studies have demonstrated improvements in metabolic outcomes by targeting specific signalling pathways in pancreatic β-cells, yet translating this knowledge into clinical practice for diabetes remains challenging. Strategies targeting UPR signalling or chaperone proteins still face multiple barriers. Given the intrinsic heterogeneity of β-cells and the time-dependent transition between adaptive and maladaptive UPR signalling, strategies should prevent the premature or delayed modulation of stress responses. The inappropriate timing of UPR activation or inhibition, through the introduction of UPR modulators, may blunt protective adaptive signalling and promote maladaptive signalling. Therefore, the stage-specific and context-dependent tuning of key UPR regulators in β-cells is essential to preserve their function within islets. However, these approaches can also exert off-target effects in non-β-cell types and other tissues. Additionally, chemical chaperones such as tauroursodeoxycholic acid (TUDCA) or 4-phenylbutyric acid (4-PBA) may ameliorate global ER stress, but their lack of β-cell specificity could limit mechanistic precision and blunt adaptive UPR signalling [135,136].

In contrast, GLP-1 receptor agonists (GLP-1RAs) and SGLT-2 inhibitors, approved by the FDA in 2005 and 2013, respectively, have shown additional health benefits in patients with kidney or cardiovascular diseases [137,138]. GLP-1RAs are well-known incretin-based treatments for diabetes, reducing blood glucose, promoting weight loss and improving overall glucose homeostasis. Notably, as a synergistic effect of improved glucose homeostasis, the dual agonism of the GLP-1/GIP receptors (tirzepatide) [139] could provide greater metabolic benefits, including superior weight loss and glucose lowering. Although multiple GLP-1RAs and tirzepatide have been approved by the FDA for obesity and T2D, the long-term effects of dual GLP-1/GIP receptor agonists on intrinsic β-cell stress signalling and UPR dynamics demand further investigation. For SGLT-2 inhibitors, given the risk of diabetic ketoacidosis [140], understanding the mechanisms by which these agents act, linked to the β-cell stress response, would be valuable for enabling them to serve as a barrier to other medications and protect β-cells. Moreover, DYRK1A inhibitors, which have been shown in preclinical studies to enhance the β-cell function by promoting β-cell proliferation [141], have also recently been suggested as potential new anti-diabetic agents, but these have not yet been tested in humans. However, optimal timing, duration and safety tests are required for the clinical application of these drugs.

Regarding the route of drug administration, research in bioengineering should be considered to improve the delivery efficiency to target tissues/cells while maintaining their inherent persistence. Stem-cell-derived β-cell transplantation aims to replace functional β-cells by modulating β-cell differentiation. Similarly, islet organoid transplantation aims to restore the islet cellular diversity, focusing on islet heterogeneity beyond β-cell replacement, thereby enabling paracrine signalling within islets and improving the neighbouring β-cell function. This would thus be beneficial for modulating the immune response and long-term engraftment.

Ultimately, mechanism-informed interventions should address β-cell heterogeneity, dynamic stress responses and species-specific differences to facilitate the systematic integration of functional islets. Additionally, an in-depth understanding of the integration among neighbouring β-cells within islets should be essential for designing interventions that preserve β-cell integrity and improve health outcomes.

## Data Availability

No new data were created or analysed in this study.

## Abbreviations

The following abbreviations are used in this manuscript:

4-PBA	4-phenylbutyric acid
14-3-3 ζ	14-3-3 ζ isoform
ATF6	Activating Transcription Factor 6
BI-1	Bax Inhibitor-1
BMDMs	Bone-marrow-derived macrophages
Ca^2+^	Calcium ion
CRTC1	CREB-regulated transcription coactivator 1
EDEM1	ER-degradation-enhancing α-mannosidase-like protein 1
ER	Endoplasmic reticulum
FFA	Free fatty acids
FFAR1	FFA receptor 1
FFAR4	FFA receptor 4
GCGR	Glucagon receptor
GLP-1RAs	GLP-1 receptor agonists
GSDMD	Gasdermin D
GSIS	Glucose-stimulated insulin secretion
GWAS	Genome-wide association studies
H3K27ac	H3K27 acetylation
HDAC	Histone deacetylase
hIAPP	Human islet amyloid polypeptide
hiPSC	Human induced pluripotent stem cell
IDF	International Diabetes Federation
IFN-γ	Interferon-γ
IL-1β	Interleukin-1β
IMC	Imaging mass cytometry
IRE1α	Inositol-Required Enzyme 1 α
mAb	Monoclonal antibody
METRNL	Meteorin-like protein
MUFA	Monounsaturated fatty acids
NGS	Next-generation sequencing
NODM	New-onset diabetes mellitus
OSGEP	O-sialoglycoprotein endopeptidase
PAIRs compound	Partial Antagonists of IRE1α RNase
PERK	PKR-like ER kinase
RIDD	Regulated IRE1-Dependent Decay
ROS	Reactive oxygen species
SASP	Senescence-associated secretory phenotype
SIRT1	Silent information regulator 1
T2D	Type 2 diabetes
TNF-α	Tumour necrosis factor-α
TRXR	Thioredoxin reductase
TUDCA	Tauroursodeoxycholic acid
TXN1	Thioredoxin 1
TXNIP	Thioredoxin-interacting protein
UPR	Unfolded protein response
